# rTMS treatment for abrogating intracerebral hemorrhage‐induced brain parenchymal metabolite clearance dysfunction in male mice by regulating intracranial lymphatic drainage

**DOI:** 10.1002/brb3.3062

**Published:** 2023-05-09

**Authors:** Yuheng Liu, Xuanhui Liu, Pengju Sun, Jing Li, Meng Nie, Junjie Gong, Anqi He, Mingyu Zhao, Chen Yang, Zengguang Wang

**Affiliations:** ^1^ Department of Neurosurgery Tianjin Medical University General Hospital Tianjin China; ^2^ Tianjin Neurological Institute, Key Laboratory of Post‐Neuroinjury Neuro‐Repair and Regeneration in Central Nervous System Ministry of Education and Tianjin Tianjin China; ^3^ Department of Neurosurgery Fuyang People's Hospital Fuyang China

**Keywords:** glymphatic system, intracerebral hemorrhage, intracranial lymphatic drainage, meningeal lymphatic vessels, repeated transcranial magnetic stimulation

## Abstract

**Background:**

The discovery of the glymphatic system and meningeal lymphatic vessels challenged the traditional view regarding the lack of a lymphatic system in the central nervous system. It is now known that the intracranial lymphatic system plays an important role in fluid transport, macromolecule uptake, and immune cell trafficking. Studies have also shown that the function of the intracranial lymphatic system is significantly associated with neurological diseases; for example, an impaired intracranial lymphatic system can lead to Tau deposition and an increased lymphocyte count in the brain tissue of mice with subarachnoid hemorrhage.

**Methods:**

In this study, we assessed the changes in the intracranial lymphatic system after intracerebral hemorrhage and the regulatory effects of repeated transcranial magnetic stimulation on the glymphatic system and meningeal lymphatic vessels in an intracerebral hemorrhage (ICH) model of male mice. Experimental mice were divided into three groups: Sham, ICH, and ICH + repeated transcranial magnetic stimulation (rTMS). Three days after ICH, mice in the ICH+rTMS group were subjected to rTMS daily for 7 days. Thereafter, the function of the intracranial lymphatic system, clearance of RITC‐dextran and FITC‐dextran, and neurological functions were evaluated.

**Results:**

Compared with the Sham group, the ICH group had an impaired glymphatic system. Importantly, rTMS treatment could improve intracranial lymphatic system function as well as behavioral functions and enhance the clearance of parenchymal RITC‐dextran and FITC‐dextran after ICH.

**Conclusion:**

Our results indicate that rTMS can abrogate ICH‐induced brain parenchymal metabolite clearance dysfunction by regulating intracranial lymphatic drainage.

## Graphical abstract

rTMS can improve brain parenchymal metabolite clearance after ICH in mice by regulating intracranial lymphatic drainage, thereby improving neurological function of ICH mice.

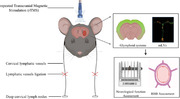

## 1 INTRODUCTION

Intracerebral hemorrhage (ICH) is one of the main causes of death and disability worldwide and can place a significant economic burden on society and families of patients (Cordonnier et al., 2018; Yao et al., 2021). The rupture of diseased blood vessels leads to the spillage of blood components into the brain parenchyma or ventricles (Cordonnier et al., 2018), whereas accumulation of extravasated blood increases the intracranial pressure (ICP) and mechanically compresses the surrounding tissues. Simultaneously, a series of secondary brain injuries also occur in the acute phase after a focal brain injury, starting within minutes and lasting for days, weeks, or even longer (Beez et al., 2017; Urday et al., 2015). Death of nerve cells and degradation of blood components stimulate glial cell activation, peripheral immune cell recruitment, and release of various cytokines and chemokines (Fu et al., 2015; Kleinschnitz et al., 2013; Mracsko & Veltkamp, 2014; Mracsko et al., 2014; Shi et al., 2018). These secondary reactions lead to blood–brain barrier (BBB) disruption, microvascular failure, and even more neuronal cell death, further aggravating brain injury and leading to impaired cognitive performance, thus affecting the long‐term prognosis of patients after ICH (Shi et al., 2019). Therefore, exploring new methods to regulate intracranial metabolite levels, inflammation, and immune responses after ICH is an important strategy for its treatment.

Iliff et al. (2012) first elucidated the potential channels for the exchange of cerebrospinal fluid (CSF) and interstitial fluid (ISF) and coined the term “glia‐lymphatic system” (glymphatic system) (Iliff et al., 2012). This system is an important channel for CSF–ISF exchange, especially for the drainage of large molecules such as low‐density lipoprotein, Tau, and beta amyloid precursor protein (Jessen et al., 2015). Louveau et al. (2015) demonstrated the presence of lymphatic vessels next to the venous sinuses of the dura mater in mice and named them meningeal lymphatic vessels (mLVs) (Louveau et al., 2015). Collectively, the glymphatic system and mLVs are referred to as the intracranial lymphatic system (Papadopoulos et al., 2020; Sun et al., 2018).

It is currently believed that the glymphatic system and mLVs play important roles in neurodegenerative diseases and the pathological progression of brain injury, cerebrovascular diseases, brain tumors, and several other diseases (Aspelund et al., 2016; Bosche et al., 2020; Chen et al., 2020; Hsu et al., 2021; Iliff et al., 2014; Liu, Wu, et al., 2021; Nikolenko et al., 2020; Welsh et al., 2016; Yanev et al., 2020). A study on patients with subarachnoid hemorrhage (SAH) showed that SAH increases Tau deposition in the brain parenchyma, decreases paravascular AQP4 polarization, and increases the number of T lymphocytes in the brain tissue, indicating dysregulated intracranial lymphatic system drainage (Pu et al., 2019). Another study showed that, compared with normal mice, transgenic mice with Alzheimer's disease had a compromised mLVs drainage and that the drainage efficiency of mLVs was significantly enhanced by transfection of vascular endothelial growth factor C in elderly mice with compromised drainage (Da Mesquita et al., 2018). Based on the available evidence, the glymphatic system and mLVs are interrelated and have important roles in regulating intracranial drainage, with the glymphatic system coordinating with mLVs to remove harmful macromolecular products from the intracranial milieu (Nikolenko et al., 2020).

Accordingly, modulating the intracranial lymphatic system to improve the prognosis of neurological disorders is considered an important treatment target for neurological diseases. Among the various nonsurgical treatments currently available, repeated transcranial magnetic stimulation (rTMS) is an important noninvasive option and further investigation into its underlying mechanisms of action in neurological diseases may reveal its therapeutic potential. The magnetic field generated by rTMS can produce electrical currents that can temporarily stimulate or inhibit specific areas of the cerebral cortex, and the clinical changes persist well beyond the period of stimulation. In fact, such effects may persist for a few minutes to hours or even days after an rTMS treatment, indicating the possibility of a sustained treatment effect (Gangitano et al., 2002; Hallett, 2000; Touge et al., 2001). For example, Lin et al. (2021) studied rTMS as a noninvasive treatment to improve the prognosis of neurological diseases by improving the clearance of metabolites in the brain parenchyma in a 5xFAD mouse model; they demonstrated significantly reduced A‐β deposition in the cortex and hippocampus (Lin et al., 2021).

In this study, we assessed the changes in the intracranial lymphatic system after ICH and the regulatory effects of rTMS on the glymphatic system and mLVs in an ICH model of male mice, providing references for clinical decision‐making.

## 2 MATERIALS AND METHODS

### 2.1 Animals

Male C57BL/6 mice (7–9 weeks old, weighing 22–24 g; Vital River Laboratory Animal Technology Co., Ltd., Beijing, China) were housed in the animal facility of Tianjin Medical University General Hospital under a 12‐h light/dark cycle in a temperature‐controlled room (20 ± 2°C) with ad libitum access to food and water. All experimental procedures involving animals were approved by the Animal Ethics Committee of Tianjin Medical University (Tianjin, China).

**FIGURE 1:**
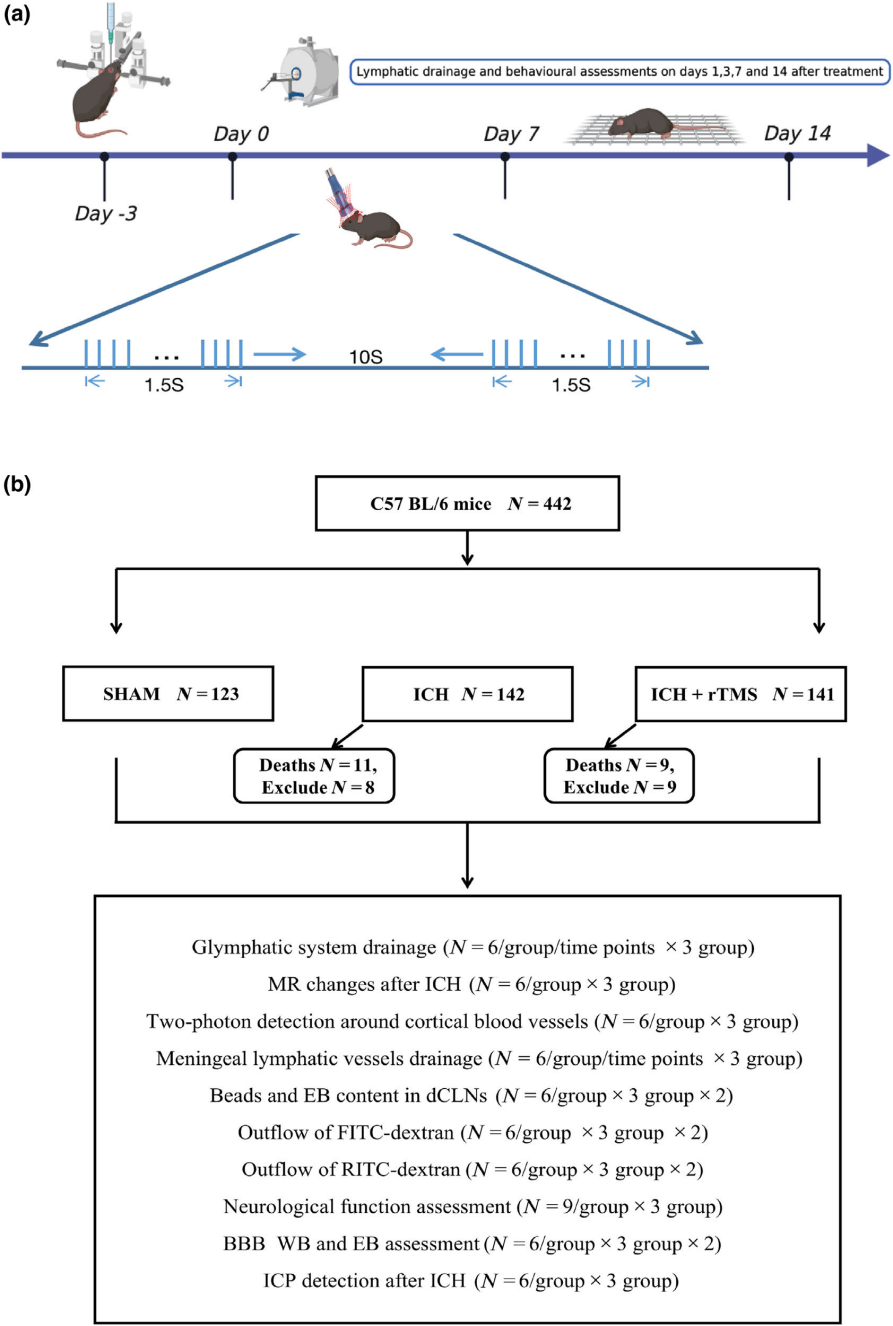
Design of the experiment. (A) rTMS treatment was started 3 days after ICH and evaluated on days 1, 3, 7, and 14 after rTMS treatment. (B) Flow chart showing the experimental protocol with the number of animals used, died, and included in the study. rTMS, repeated transcranial magnetic stimulation; ICH, intracerebral hemorrhage.

The experimental mice were divided into three groups: Sham, ICH, and ICH+rTMS. Collagenase was administered to the brains of ICH and ICH+rTMS group mice to establish the ICH model. The Sham group mice were injected with saline to simulate the micro‐injection needle injury. Mice in the ICH+rTMS group first received the rTMS 72 h after ICH and were subjected to the treatment for a total of seven consecutive days; each mouse was subjected to a total of 1560 stimulations (20 min) each day (Figure 1A). The ICH group mice underwent the same procedure but were fixed in an area outside the range of the magnetic coil.

### 2.2 ICH model

Mice were anesthetized by intraperitoneal injection of a mixture of ketamine (100 mg/kg) and xylazine (10 mg/kg). Since the depth of anesthesia has an important effect on intracranial lymphatic drainage, during anesthesia of our study, the vital signs of the mice were stably maintained—respiration rate, 50–60 breaths per minute; SO_2_ saturation, approximately 98%–100%; heart rate, nearly 300–370 beats per minute; body temperature, 36.5–37.5°C. The hair on the dorsal side of the head was then removed, the head was fixed in a stereotactic frame, and an incision was made to expose the skull and bregma. A round hole (diameter, approximately 0.2 mm) was drilled 0.5 mm in front of the bregma and 2.3 mm on the right side of the midline. A micro‐injection needle (33‐G flat needle) was inserted 3.5 mm vertically in the center of the hole, and a micro‐injection pump (Shenzhen Ruiwode Life Technology Co., China) was then used to inject 0.42 μL collagenase (0.5 U/μL; Sigma C5138) into the brain at an average speed of 0.5 μL/min. The needle was left undisturbed for 10 min after injection and then slowly pulled back upward, and the skin incision was then sutured after disinfection. Thereafter, the mice were allowed to recover in a warm and ventilated environment. Twenty‐four hours after collagenase injection, mice without hemiplegia were excluded from the experiment.

### 2.3 Repetitive transcranial magnetic stimulation

Mice were immobilized using a fabricated fixation device (Lin et al., 2021). It was fixed on the foam plate using a simple sleeve; a bevel gap was left at the top of the sleeve to expose the head of the animal, and the tightness of the fixture was adjusted using nylon cable ties to ensure that the sleeve limited the mouse's activity to the greatest extent without affecting its breathing, and that there was no excessive struggle‐related resistance. A magnetic stimulator (CCY‐IA, Wuhan YiRuiDe Medical Equipment Co., Wuhan, China) connected to a circular coil (diameter: 6.5 cm, maximum output strength: 3.64 T) was used for the rTMS. Briefly, during rTMS stimulation, the center of the stimulation coil was placed close to the head of the mouse (aligned with its center on the midline, equidistant between the bregma and lambda sutures along the longitudinal body axis; the coil–scalp distance was 1.0 cm) (Ma et al., 2014).

In order to select the appropriate rTMS parameters, we took into account a large number of previous studies (Chen et al., 2019; Cui et al., 2019; Grehl et al., 2015; Lin et al., 2021; Ma et al., 2019; Poh et al., 2019; Wang et al., 2015; Zhu et al., 2020), and compared with the low frequency, the high‐frequency (10 Hz) rTMS treatment showed better improvement effects on the cognitive function and metabolites clearance. Therefore, in our study, we choose the rTMS parameters from Poh's previous research (Poh et al., 2019). The parameters of the stimulation received by the mice were as follows: 25% maximum output intensity, approximately 0.91 T; stimulation frequency, 10 Hz; stimulation time, 1.5 s; time interval, 10 s; total number of stimulations, 1560 (Figure 1A).

Currently, the timing of rTMS treatment for ICH varies across clinical studies, ranging from 3 to 90 days after stroke (Du et al., 2016; Huang et al., 2018; Matsuura et al., 2015). Considering mice limb recovery after ICH, we chose to subject them to the rTMS intervention 3 days after ICH to obtain more beneficial improvements.

### 2.4 Assessment of glymphatic function

Glymphatic system function was evaluated as previously described (Da Mesquita et al., 2018; Hou et al., 2022; Iliff et al., 2012; Yang et al., 2013). On days 1, 3, 7, and 14 after rTMS treatment, the mice were anesthetized and had their head fixed in a stereotactic frame. Thereafter, 10 μL of rhodamine B isothiocyanate (RITC)‐dextran (Sigma R9379; molecular weight [MW] = 70 kDa; 25 μg/mL) was infused into the subarachnoid CSF via the cisterna magna at a rate of 2 μL/min for 5 min (total volume, 10 μL). The mice were sacrificed 20 min after the injection; whole‐brain tissue was extracted and fixed overnight in 4% paraformaldehyde (PFA) and then embedded in optimal cutting temperature compound (OCT). Subsequently, 100‐μm slices were prepared using a freezing microtome and observed using a fluorescence microscope. RITC‐dextran influx was quantified by a blinded investigator using ImageJ as described previously (Iliff et al., 2012). The slices were imaged at 1.25× using a fluorescence microscope, and montages were generated. To evaluate RITC‐dextran coverage, the color channels were separated, and the image background was subtracted. The whole‐brain area in each slice was manually outlined and uniformly thresholded, and the thresholded area was then calculated and expressed as a percentage of the overall slice area. A weighted average of RITC‐dextran between nine random brain slices was calculated for a single animal (Figure S1).

### 2.5 Enhanced magnetic resonance imaging

Gd‐DTPA remains in the extracellular space and cannot penetrate nerve cells, making it a good indicator for observing ISF flow (Iliff, Lee, et al., 2013). Therefore, Gd‐DTPA and magnetic resonance imaging (MRI) scanning were used to assess the characteristics of ISF drainage and evaluate lymphatic system drainage. On day 7 after rTMS treatment, mice in each group were subjected to enhanced MRI‐based examination. Briefly, the mice were anesthetized using 2% isoflurane and 0.8 L/min O_2_ and with the head fixed in the stereotactic frame. During anesthesia, the vital signs of the mice were stably maintained—respiration rate, 50–60 breaths per minute; SO_2_ saturation, approximately 98%–100%; heart rate, approximately 300–370 beats per minute; body temperature, 36.5–37.5°C (Iliff, Lee, et al., 2013). Thereafter, 5 μL Gd‐DTPA was infused into the cisterna magna of the mice at a rate of 5 μL/min using the same method mentioned above. The needle was slowly removed 10 min after injection, and axial and sagittal MRI scans were obtained 15, 30, 60, and 90 min after injection. A 9.4‐T MRI system (Brooke BioSpec 94/30 USR) with an eight‐channel coil was used to rapidly acquire T1‐weighted gradient echo (MP‐RAGE) sequence brain images of the animals. The acquisition parameters were as follows: echedelay time (ET) = 3.7 ms, repetition time (RT) = 1500 ms, reversal angle = 12°, reversal time = 900 ms. After scanning, ImageJ was used to analyze the concentration of Gd‐DTPA in the brain parenchyma of mice at 15, 30, 60, and 90 min after injection.

### 2.6 Two‐photon detection

On day 7 after rTMS treatment, the mice were anesthetized, and the hair on the dorsal side of the head was removed. A window approximately 3 mm in diameter was drilled in the parietal bone area and sealed with a glass coverslip to visualize the cerebrovascular system. Each mouse was then fixed in a stereotactic frame, and 5 μL of FITC‐dextran (Sigma, 46944; MW = 2000 kDa) was injected into the cisterna magna at a rate of 5 μL/min using a microsyringe pump. The needle was removed 10 min after the injection, and the mouse tail vein was then injected with 50 μL of RITC‐dextran (Sigma R9379; MW = 70 kDa). After the injection, the OLYMPUS two‐photon imaging system (OLYMPUS FV30, Tokyo, Japan) was used to image the cortex with a 25× water‐immersed lens at an excitation wavelength of 900 nm. The cortical vascular arteries and veins were differentiated according to their morphology. Localization of arteries in the damaged hemispheric cortex and imaging of the paravascular region were performed to assess the diffusion of the tracers. Two arteries were assessed in each mouse; for each vessel, we randomly selected three regions of interest for fluorescence intensity measurements. The intensity was quantified using ImageJ software. A weighted average of RITC‐dextran fluorescence in the whole‐region tracers was calculated between six random slices for a single animal (Figure S1) (He et al., 2017; Liu, Zhu, et al., 2021).

### 2.7 Assessment of meningeal lymphatic function

The function of the meningeal lymphatics was assessed as previously described (Bolte et al., 2020; Da Mesquita et al., 2018). On days 1, 3, 7, and 14 after rTMS treatment, the mice were anesthetized, and their heads were fixed in the stereotactic frame. Thereafter, 5 μL of Beads (Invitrogen, F8813) was injected into the cisterna magna at a rate of 2 μL/min using a microsyringe pump. To prevent reverse flow after needle removal, the needle was left undisturbed for 10 min after injection and then slowly pulled back upward. The mouse was sacrificed 10 min after the needle was removed and perfused with ice phosphate‐buffered saline (PBS), and the meninges were extracted. The meninges were fixed in the dark using 4% PFA at 4°C overnight. The meninges were then incubated for 1.5 h at room temperature in a permeabilization and blocking solution containing 2% bovine serum albumin, 2% goat serum, 0.2% Triton X‐100, and 0.05% Tween 20. The meninges were then incubated overnight at 4°C with primary antibody (anti‐LYVE‐1, Abcam, ab14917, 1:500). After rinsing with PBS, fluorescence‐labeled secondary antibody was added and incubated at room temperature for 1 h, followed by staining with DAPI. A fluorescence microscope was used to acquire images at 1.25×, and montages were then generated. To evaluate tracer coverage, the color channels were separated, the image background subtracted and uniformly thresholded, and the thresholded area was calculated and expressed as a percentage of the overall slice area. ImageJ software was used for quantification.

Specially, on day 7 after rTMS treatment, another three groups of mice were subjected to bilateral deep cervical lymph nodes (dCLNs) extraction 30 min after Beads injection. The dCLNs were embedded in OCT and continuously sectioned (thickness, 10 μm) using a freezing microtome (CM 1950; Leica Biosystems, Deer Park, IL, USA). And then, immunofluorescence was performed and analyzed using the same method mentioned above.

### 2.8 Evans blue colorimetric method for deep cervical lymph nodes

On day 7 after rTMS treatment, the mice were anesthetized, their heads were fixed in a stereotactic frame, and 5 μL of 2% Evans blue (EB) (E2129; Sigma–Aldrich, St. Louis, MO, USA) was injected into the cisterna magna at a rate of 2 μL/min. After needle extraction, the mice were fixed in the supine position and the neck tissue was isolated to expose the bilateral dCLNs. EB drainage of the bilateral dCLNs was observed and photographed microscopically in real time using Olympus SZX‐7, 2 h after the injection. After that, the bilateral dCLNs were extracted and placed in tubes, and after adding 200 μL of formamide to each tube, samples were extracted in a 60°C water bath for 24 h. The tubes were then centrifuged at room temperature at a speed of 4000 × *g* for 15 min, and 80 μL of the supernatant was added to a 96‐well plate. The reference scale was obtained using EB diluted with formamide in a concentration range of 25.6–0.4 μg/mL. The optical density (OD) value was measured at a wavelength of 610 nm using a microplate reader. Finally, Microsoft Excel was used to calculate the actual EB concentration based on the standard curve.

### 2.9 Intraparenchymal injections

As described previously (Da Mesquita et al., 2018; Iliff et al., 2012), metabolite clearance from the intracranial lymphatic system was assessed by analyzing fluorescent tracers injected into the brain parenchyma. On day 7 after rTMS treatment, the mice were anesthetized and fixed in a stereotactic frame. A round hole was then drilled 0.5 mm in front of the bregma and 2.3 mm on the right side of the midline. A micro‐injection needle (33‐G flat needle) was inserted 3.5 mm vertically in the center of the hole, and a micro‐injection pump (Shenzhen Ruiwode Life Technology Co., China) was then used to inject 2 μL of RITC‐dextran (Sigma R9379; MW = 70 kDa) and FITC‐dextran (Sigma, 46944; MW = 2000 kDa) into the brain at an average speed of 1 μL/min. The needle was left undisturbed for 10 min after injection and then slowly pulled back upward. Thirty minutes later, the intact brain tissue was extracted and fixed overnight in the dark using 4% PFA. The samples were then transferred to 15% or 30% sucrose for dehydration. Thereafter, the brain tissue was embedded in OCT, and 100‐μm slices were prepared using a freezing microtome. The remnant fluorescent tracers in brain slices were imaged and analyzed using a fluorescence microscope at 1.25×, and image montages were generated. The fluorescence tracers were quantified using ImageJ. To evaluate tracer coverage, the color channels were separated, and the image background subtracted. The whole‐brain area in each slice was manually outlined and uniformly thresholded, and the thresholded area was then calculated and expressed as a percentage of the overall slice area. The weighted average of the thresholded area was calculated for the brain between six random slices for a single animal, resulting in a single paired biological replicate (Liu, Yan, et al., 2020).

### 2.10 Bilateral cervical lymphatic vessel ligation

By ligating the bilateral cervical lymphatic vessels, we assessed changes in the clearance of the fluorescent tracers within the brain parenchyma. For this analysis, we set up three groups: ICH, Control, and Ligation; mice in all three groups received collagenase injection to establish a model of ICH. The Ligation group underwent bilateral deep cervical lymphatic vessels ligation 24 h before collagenase injection, and the Control group underwent the same surgical procedure but without actual ligation of the lymphatic vessels. Mice in the Ligation and Control groups were given rTMS treatment as mentioned above.

For lymphatic vessel ligation, the Ligation group mice were anesthetized, the neck hair was removed, and a skin incision approximately 2 cm long was made along the midline of the neck. The fascia of the neck was separated layer by layer; the thyroid gland was lifted to the side of the head, exposing the anterior cervical and sternocleidomastoid muscles; and the lymphatic vessels and lymph nodes in the deep neck were separated. All deep cervical lymphatic vessels were carefully ligated using a 9‐0 sterile nylon thread (care was taken to avoid the cervical blood vessels and nerves), followed by ligation of the deep lymphatic vessels of the other side in the same way, restoring the position of the thyroid tissue. The Control group mice underwent the same procedure, except for ligation of the bilateral lymphatic vessels.

### 2.11 Assessment of neurological function

On days 1, 3, 7, and 14 after rTMS treatment, neurological function of the mice was assessed. Mouse exercise capacity was evaluated using a rotarod apparatus (YLS‐4C; Yima Optoelec Co., Ltd., Beijing, China). Mice were given 3 min for acclimation to the instrument rotating at 10 rpm. Thereafter, the instrument accelerated from 0 to 40 rpm for 5 min, and the fall time (latency time) was recorded for each mouse. If mice could run in a normal posture for >5 min, the fall time was calculated as 300 s. Each mouse was assessed three times at each time point, with a 30‐min interval, so that they were adequately rested. The average latency time of the three tests was taken as the latency for that time point.

The foot fault test was used to assess sensorimotor coordination. Mice were placed on an elevated grid surface (30 × 35 × 31 cm [*l* × *w* × *h*]) with a 2.5‐cm^2^ grid opening, and scores for 1 min were recorded by a blinded experimenter. The total number of steps and number of foot faults (when the animals misplaced a forepaw or hindpaw such that it fell through the grid) were counted (Cheng et al., 2021; Zhang et al., 2019).

### 2.12 BBB damage

BBB integrity was assessed using EB extravasation according to a previous study (Liu et al., 2022; O'Connor et al., 2005). On day 7 after rTMS treatment, the mice were anesthetized, and 100 μL of 2% EB (E2129; Sigma–Aldrich) was injected into the tail vein. Two hours after injection, the mice were euthanized and immediately perfused with cold PBS. Brain tissue samples of the injured cerebral hemisphere were cut into pieces in a tube. After adding 1 mL of formamide to each tube, samples were extracted in a 60°C water bath for 24 h. The tubes were then centrifuged at room temperature at a speed of 4000 × *g* for 15 min, and 200 μL of the supernatant was added to a 96‐well plate. The reference scale was obtained using EB diluted with formamide in a concentration range of 25.6–0.4 μg/mL. The OD value for each well was spectroscopically detected at 610 nm. Based on the reference values, the EB concentration of each supernatant sample was calculated.

### 2.13 Assessment of BBB‐related protein expression

On day 7 after rTMS treatment, the mice were anesthetized, and the injured cerebral hemisphere tissue was obtained. Total protein was extracted using RIPA protein lysis buffer and 1% Phenylmethanesulfonyl fluoride (PMSF). The protein sample was separated by 10% SDS‐PAGE at 120 V for 90 min, and the separated proteins were transferred onto polyvinylidene fluoride (PVDF) membranes. These PVDF membranes were then blocked, cut into bands, and incubated with primary antibodies against ZO‐1 (Thermo Fisher, 61–7300, 1:1000), claudin‐5 (Thermo Fisher, 35–2500, 1:1000), and β‐actin (Proteintech, 66009, 1:5,000) overnight at 4°C on a shaker. After washing three times, the membrane bands were incubated with an Horseradish Peroxidase (HRP)‐coupled secondary antibody for 1 h at room temperature. A ChemiDoc Imaging System (Bio‐Rad, Hercules, CA, USA) was used to detect the enhanced chemiluminescence (ECL) chemiluminescence, and ImageJ was used for quantifying the intensity.

### 2.14 Data analysis

The assumption of normally distributed data was checked using the Shapiro–Wilk test. One‐way analysis of variance was used to compare differences among the groups, followed by Fisher's least significant difference test for post hoc comparisons. GPower (ver. 3.1.9.7; Heinrich‐Heine‐Universität, Düsseldorf, Germany) was used to estimate exact sample sizes. The study power was set at 80% and the alpha value at .05. Data were expressed as the mean ± standard deviation values. A *p*‐value of <.05 was considered statistically significant. All statistical analyses were performed in GraphPad Prism (version 9.0).

## 3 RESULTS

### 3.1 rTMS improved glymphatic system drainage after ICH

**FIGURE 2:**
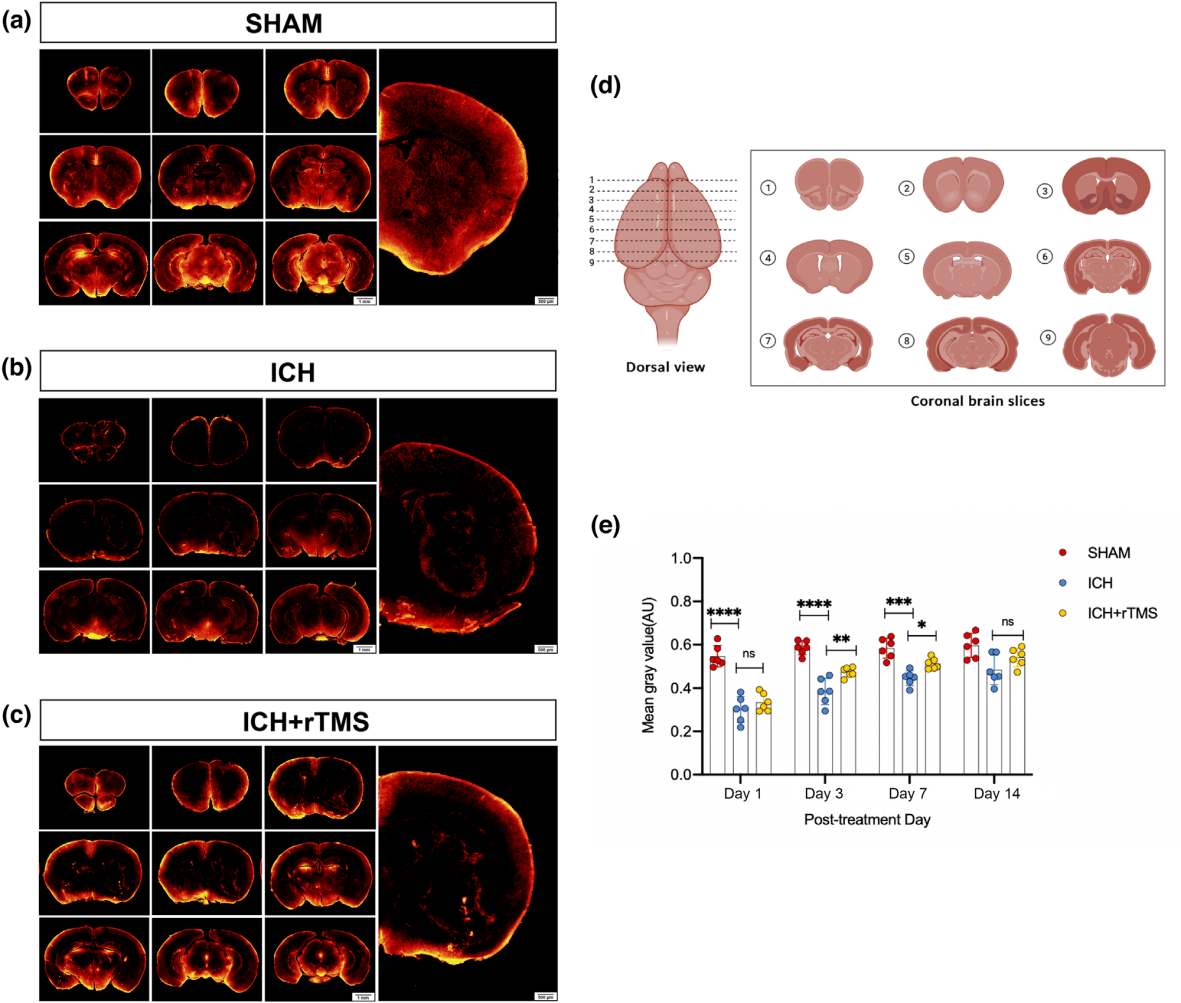
Functional changes of glymphatic system after ICH. Panels A–C show the distribution of RITC‐dextran in the brain of each group on day 7 after treatment; panel D shows the region of brain sections; panel E shows that, compared with the Sham group, the proportion of RITC‐dextran in the brain decreased on days 1, 3, and 7 after rTMS treatment in the ICH group (day 1: *F*_(2, 15)_ = 41.67, *n* = 6, *p* < .0001; day 3: *F*_(2, 15)_ = 34.95, *n* = 6, *p* < .0001; day 7: *F*_(2, 15)_ = 21.61, *n* = 6, *p* < .0001), and rTMS can improve the distribution of RITC‐dextran in brain, and there is a statistical difference compared with the ICH group on days 3 and 7 after rTMS treatment (day 3: *F*_(2, 15)_ = 34.95, *n* = 6, *p* < .0001; day 7: *F*_(2, 15)_ = 21.61, *n* = 6, *p* = .0161). *p < .05; **p < .01; ***p < .001; ****p < .0001. rTMS, repeated transcranial magnetic stimulation; ICH, intracerebral hemorrhage.

The function of the glymphatic system was evaluated after rTMS treatment on days 1, 3, 7, and 14. RITC‐dextran was injected into the cisterna magna, and the proportion of residual RITC‐dextran was calculated using fluorescence microscopy images. The proportions of RITC‐dextran in the brain in the ICH group on days 1, 3, and 7 were lower than those in the Sham group (*p* < .05) (Figure 2E). Thus, assessment of the effects of rTMS on glymphatic system drainage after ICH showed that rTMS improved the distribution of RITC‐dextran in the brain. A statistical difference was observed between the ICH group and ICH+rTMS group 3 and 7 days after rTMS treatment (*p* < .05) (Figure 2E).

### 3.2 rTMS improved the diffusion of Gd‐DTPA and RITC‐dextran in the cortex after ICH on day 7 after treatment

**FIGURE 3:**
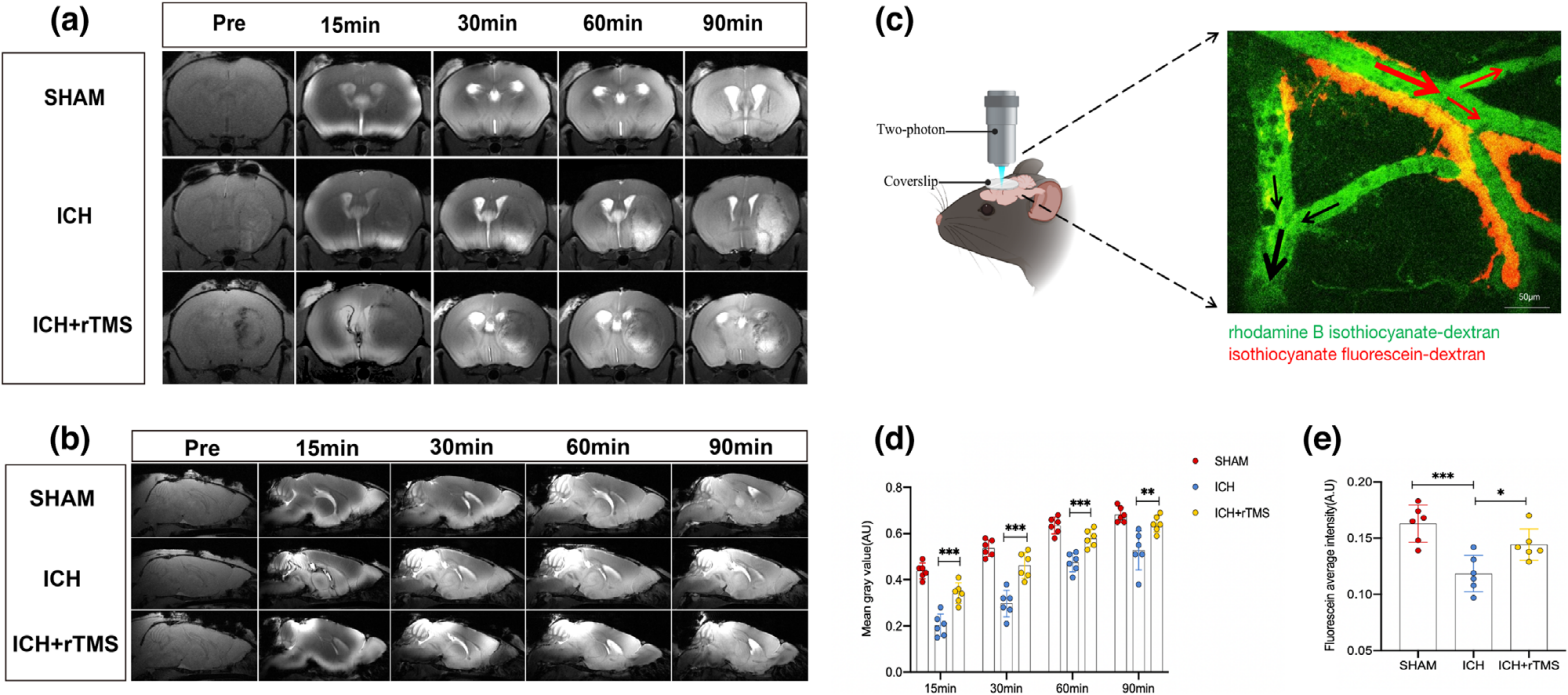
rTMS improves diffusion of Gd‐DTPA and FITC‐dextran in the cortex 7 days after treatment. Panels A and B show the axial and sagittal serial imaging of Gd‐DTPA distribution at different time points after Gd‐DTPA injection; panel C shows the distribution of perivascular RITC‐dextran in the cortex under two‐photon microscopy (the red arrow represents arterial blood flow and the black arrow represents venous blood flow); panel D shows that, compared with the Sham group, the inflow of Gd‐DTPA into the brain decreased after ICH, and the inflow of Gd‐DTPA into the brain improved in the ICH+rTMS group compared with that in the ICH group (15 min: *F*_(2, 15)_ = 46.33, *n* = 6, *p* = .0001; 30 min: *F*_(2, 15)_ = 35.19, *n* = 6, *p* = .0001; 60 min: *F*_(2, 15)_ = 27.16, *n* = 6, *p* < .0001; 90 min: *F*_(2, 15)_ = 12.43, *n* = 6, *p* = .0075); panel E shows that, compared with the Sham group, cortical arterial perivascular FITC‐dextran decreased after ICH (*F*_(2, 15)_ = 12.29, *n* = 6, *p* = .0005), and the amount of perivascular FITC‐dextran could be increased after rTMS treatment (*F*_(2, 15)_ = 12.29, *n* = 6, *p* = .0303). *p < .05; **p < .01; ***p < .001; ****p < .0001. rTMS, repeated transcranial magnetic stimulation; ICH, intracerebral hemorrhage.

Continuous MRI scanning after Gd‐DTPA injection into the cisterna magna indicated that brain Gd‐DTPA levels 7 days after treatment were significantly lower at different time points than those in the Sham group. Compared with the ICH group, the ICH+rTMS group had significantly higher brain Gd‐DTPA levels on day 7 after rTMS treatment (15, 30, 60, and 90 min; *p* < .05) (Figure 3A,B,D).

RITC‐dextran inflow around the cortical arteries was evaluated using in vivo two‐photon imaging. Compared with that in the Sham group, the inflow of RITC‐dextran around the cortical arteries was significantly lower in the ICH group (*p* < .05). Furthermore, the inflow of RITC‐dextran around the cortical arteries in the ICH+rTMS group was significantly higher than that in the ICH group on day 7 after rTMS treatment (*p* < .05) (Figure 3C,E).

### 3.3 rTMS enhanced the drainage function of mLVs after ICH

**FIGURE 4:**
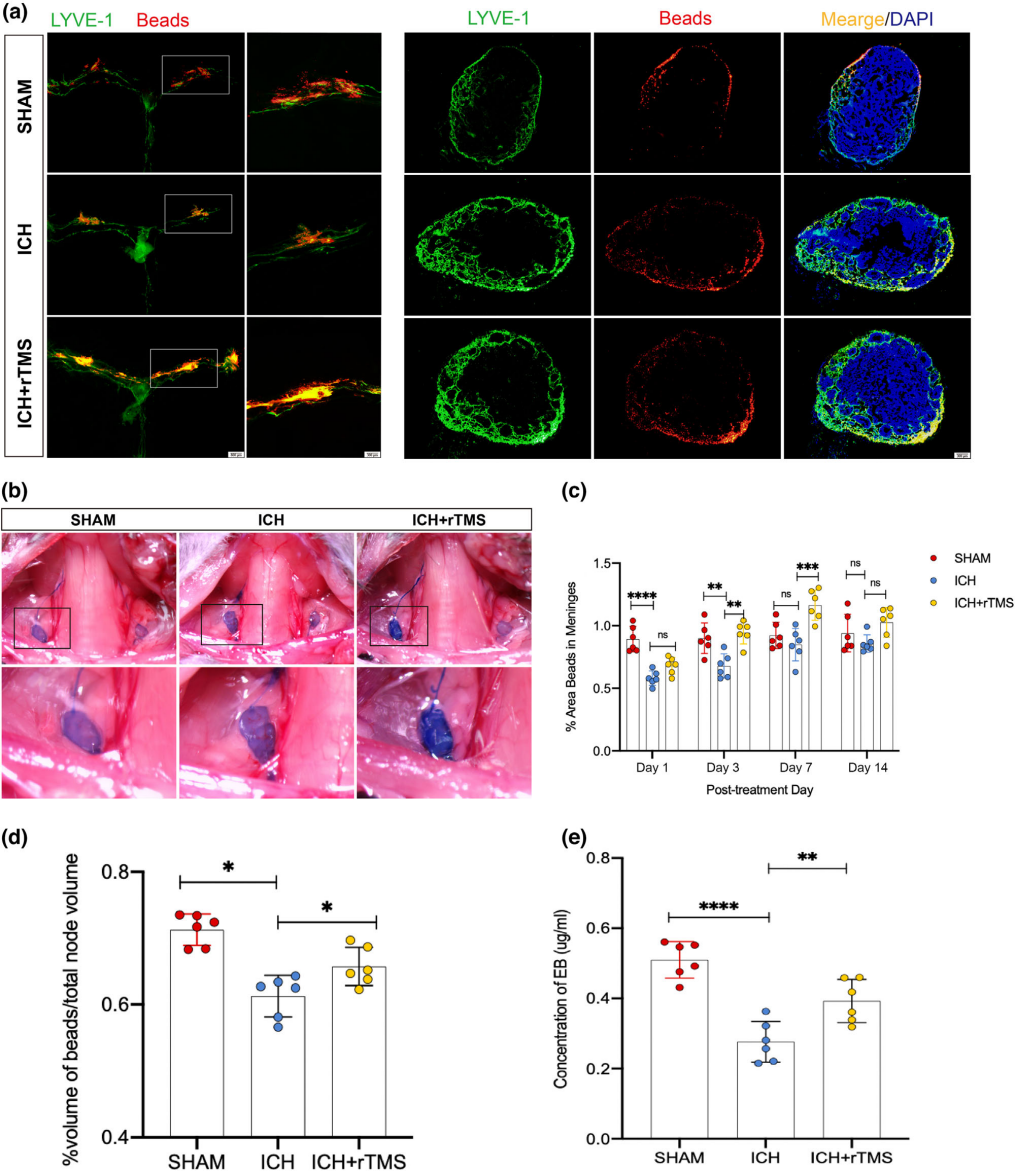
Functional changes of meningeal lymphatic vessels after ICH. Panel A shows the distribution of Beads in the meningeal lymphatic vessels or dCLNs of each group on day 7 after treatment; panel B shows the distribution of EB in the dCLNs of each group on day 7 after treatment; panel C shows that, compared with the Sham group, the proportion of Beads in mLVs decreased on days 1 and 3 after rTMS treatment in the ICH group (day 1: *F*_(2, 15)_ = 25.20, *n* = 6, *p* < .0001; day 3: *F*_(2, 15)_ = 11.33, *n* = 6, *p* = .0057), and the percentage of Beads in the mLVs in the ICH+rTMS group increased compared that with the ICH group, and the results showed a statistically significant difference in the days 3 and 7 after treatment (day 3: *F*_(2, 15)_ = 25.20, *n* = 6, *p* = .0013; day 7: *F*_(2, 15)_ = 11.33, *n* = 6). Panels D and E show that, on day 7 after treatment, compared with the Sham group, the content of Beads or EB in dCLNs decreased in the ICH group (Beads: *F*_(2, 15)_ = 19.11, *n* = 6, *p* = .0373; EB: *F*_(2, 15)_ = 24.92, *n* = 6, *p* = .0083), and after rTMS treatment, the content of Beads or EB in dCLNs increased comparing with that in the ICH group (Beads: *F*_(2, 15)_ = 19.11, *n* = 6, *p* = .0100; EB: *F*_(2, 15)_ = 24.92, *n* = 6, *p* = .0078). *p < .05; **p < .01; ***p < .001; ****p < .0001. EB, Evans blue; dCLNs, deep cervical lymph nodes; rTMS, repeated transcranial magnetic stimulation; ICH, intracerebral hemorrhage.

The drainage function of mLVs was evaluated 1, 3, 7, and 14 days after rTMS treatment. The results showed that, compared with that in the Sham group, the proportion of Beads in the mLVs in the ICH group was significantly lower on days 1 and 3 (*p* < .05). Moreover, the proportion of Beads in the mLVs in the ICH+rTMS group was significantly higher than that in the ICH group on days 3 and 7 (*p* < .05) (Figure 4A,C).

### 3.4 rTMS increased Beads/EB drainage to the deep cervical lymph nodes on day 7 after treatment

On day 7 after rTMS treatment, Beads content in the bilateral dCLNs was assessed 30 min after they were injected into the cisterna magna. Compared with that in the Sham group, Beads content in the dCLNs was lower than that in the ICH group, and Beads content in the dCLNs was higher in the ICH+rTMS group than that in the ICH group (*p* < .05) (Figure 4A,D).

Similarly, on day 7 after rTMS treatment, EB content in the bilateral dCLNs was assessed 2 h after it was injected into the cisterna magna. The results showed that the outflow of EB in the bilateral dCLNs was lower in the ICH group than that in the Sham group (*p* < .05). Compared with that in the ICH group, the EB level in the bilateral dCLNs was significantly higher in the ICH+rTMS group (*p* < .05) (Figure 4B,E).

### 3.5 rTMS improved the clearance of brain parenchymal fluorescent tracers on day 7 after treatment

**FIGURE 5:**
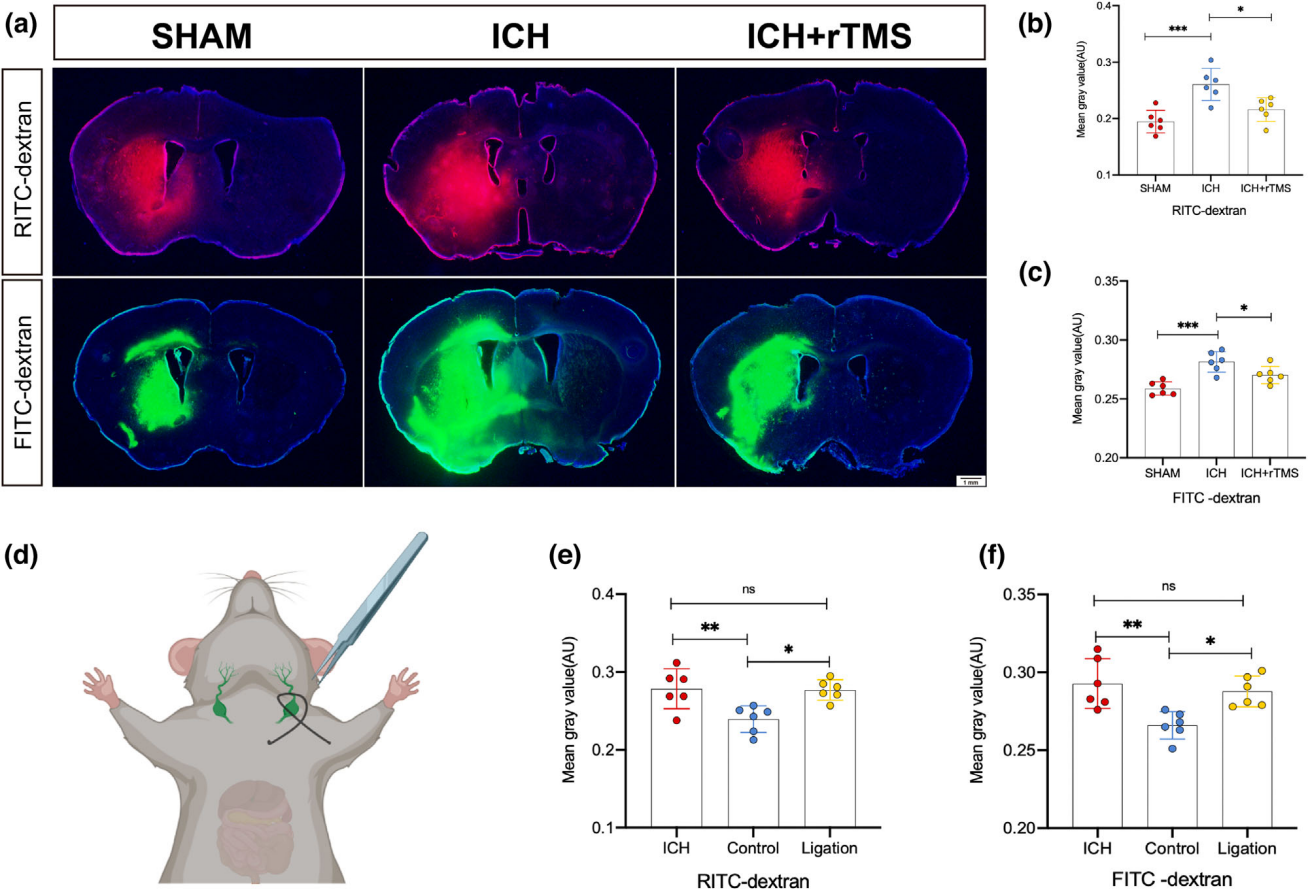
rTMS improves the outflow of RITC‐dextran and FITC‐dextran in brain parenchyma on day 7 after rTMS treatment. Panel A shows the outflow of RITC‐dextran and FITC‐dextran of each group on day 7 after treatment. Panels B and C show that compared with the Sham group, the outflow of RITC‐dextran and FITC‐dextran was weakened after ICH (RITC‐dextran: *F*_(2, 15)_ = 12.48, *n* = 6, *p* = .0005; FITC‐dextran: *F*_(2, 15)_ = 14.21, *n* = 6, *p* = .0002), and the outflow of RITC‐dextran and FITC‐dextran in the brain parenchyma could be improved after rTMS treatment (RITC‐dextran: *F*_(2, 15)_ = 12.48, *n* = 6, *p* = .0123; FITC‐dextran: *F*_(2, 15)_ = 14.21, *n* = 6, *p* = .0474). Panel D shows the process of lymphatic vessel ligation; panels E and F show the function of rTMS for improving clearance of fluorescent tracers in the brain parenchyma after ICH disappears (RITC‐dextran: *F*_(2, 15)_ = 7.818, *n* = 6, *p* = .9901; FITC‐dextran: *F*_(2, 15)_ = 8.525, *n* = 6, *p* = .7537). *p < .05; **p < .01; ***p < .001; ****p < .0001. rTMS, repeated transcranial magnetic stimulation; ICH, intracerebral hemorrhage.

Assessment of the outflow of intraparenchymal fluorescent tracers (Figure 5A) showed that the brain parenchyma clearance of RITC‐dextran and FITC‐dextran in the ICH group was significantly lower than that in the Sham group (RITC‐dextran, *p* < .05; FITC‐dextran, *p* < .05). Additionally, rTMS treatment significantly improved the clearance of RITC‐dextran and FITC‐dextran in the brain parenchyma after ICH (RITC‐dextran, *p* < .05; FITC‐dextran, *p* < .05) (Figure 5A–C).

### 3.6 rTMS improved fluorescent tracers’ accumulation in brain parenchyma by regulating intracranial lymphatic drainage

We blocked the function of intracranial lymphatic drainage by ligating the bilateral cervical lymphatic vessels and assessed the clearance of RITC‐dextran and FITC‐dextran injected into the brain parenchyma to clarify the role of the intracranial lymphatic drainage in the clearance of brain parenchymal fluorescent tracers. Compared with that in the ICH group, RITC‐dextran and FITC‐dextran accumulation in the intracranial space was significantly reduced after rTMS treatment in the Control group, in which deep cervical lymphatic vessel function was intact (RITC‐dextran, *p* < .05; FITC‐dextran, *p* < .05). However, compared with those in the ICH group, there were no significant differences in RITC‐dextran and FITC‐dextran levels in brain parenchyma after rTMS treatment in the Ligation group, in which the deep cervical lymphatic vessels was ligated (RITC‐dextran, *p* > .05; FITC‐dextran, *p* > .05) (Figure 5E,F).

### 3.7 rTMS improved neurological and motor function after ICH in mice

**FIGURE 6:**
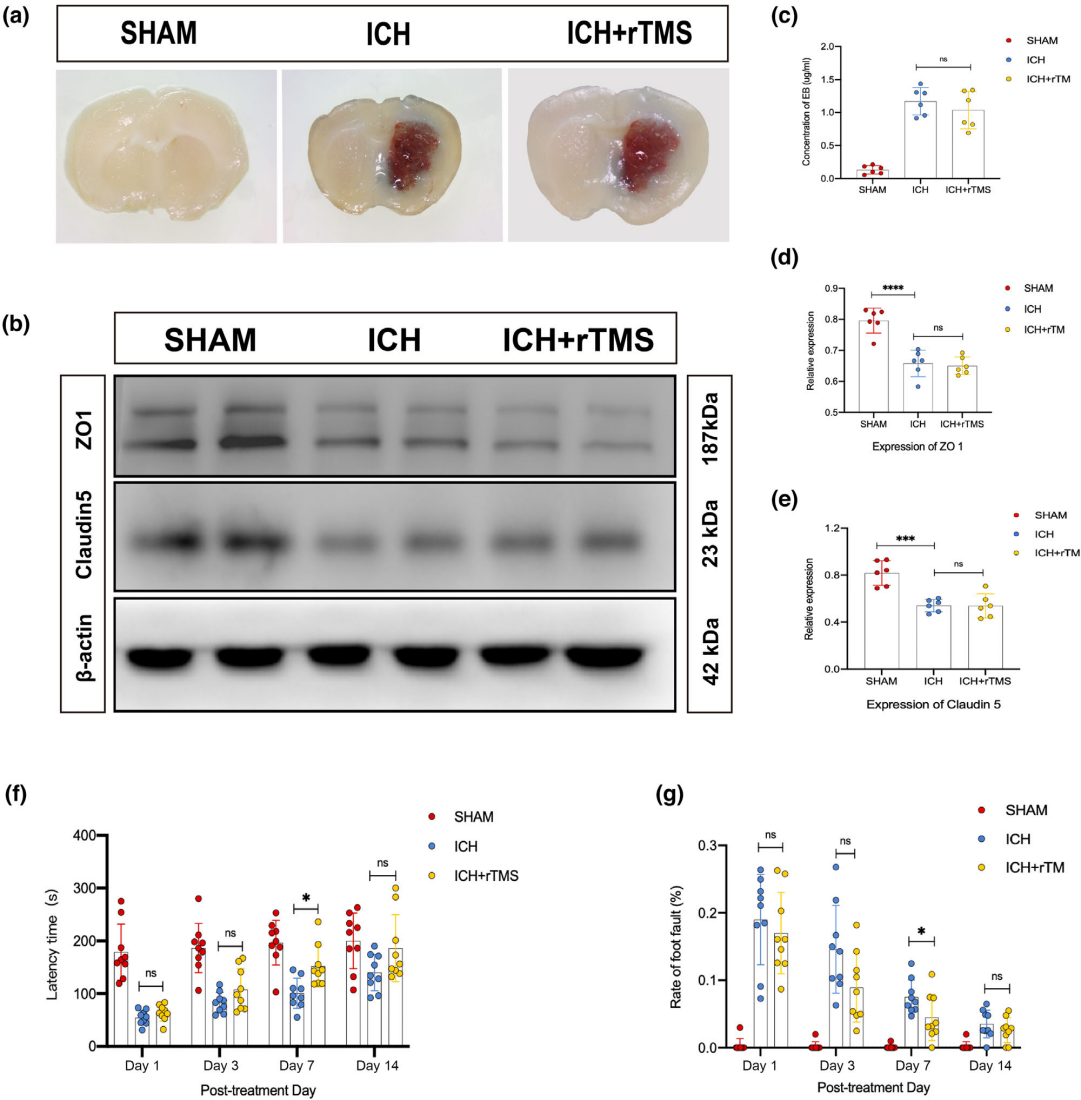
rTMS does not aggravate the damage of blood–brain barrier on day 7 after rTMS treatment and it can improve the neurological function after ICH. Panel A shows the EB permeability of each group on day 7 after treatment; panel B shows the expression of ZO‐1 and claudin‐5 of each group on day 7 after treatment. Panels C and D show that by analyzing the expression of ZO‐1 and claudin‐5 in brain tissue, there was no statistical difference between ICH and ICH+rTMS groups (ZO‐1: *F*_(2, 15)_ = 0.1443, *n* = 6, *p* = .9369; claudin‐5: *F*_(2, 15)_ = 0.8807, *n* = 6, *p* = .9987); panel E shows there was no statistical difference in the amount of EB permeability in the ICH+rTMS group compared with that in the ICH group (*F*_(3, 20)_ = 56.15, *n* = 6, *p* = .5742). Panel F shows the latency time in the rotarod test of the ICH+rTMS group mice was longer than that in the ICH group, and the difference was statistically significant on day 7 after rTMS treatment (*F*_(2, 24)_ = 13.05, *n* = 9, *p* = .0404). Panel G shows the error rate of mice in the ICH+rTMS group was lower than that in the ICH group in foot fault test, and the difference was statistically significant on day 7 after rTMS treatment (*F*_(2, 24)_ = 19.71, *n* = 9, *p* = .0396). *p < .05; **p < .01; ***p < .001; ****p < .0001. rTMS, repeated transcranial magnetic stimulation; ICH, intracerebral hemorrhage.

On days 1, 3, 7, and 14 after rTMS treatment, the neurological and motor functions of the mice were assessed. In the rotarod test, ICH+rTMS group mice had a higher latency time than ICH group mice on day 7 (*p* < .05) (Figure 6F). In the foot fault test, the error rate of mice in the ICH+rTMS group was significantly lower than that in the ICH group on day 7 (*p* < .05) (Figure 6G).

### 3.8 rTMS did not aggravate BBB disruption in ICH mice on day 7 after treatment

In order to evaluate the effect of rTMS on the BBB and exclude the influence of the BBB on the glymphatic system in this study, BBB integrity was assessed based on the level of EB leakage on day 7 after rTMS treatment. The results showed that there was no significant difference in the level of EB permeability in the ICH+rTMS group compared with that in the ICH group (*p* < .05) (Figure 6A,C). Furthermore, there were no significant differences in the expression levels of ZO‐1 and claudin‐5 in brain tissue between the two groups (*p* > .05) (Figure 6B,D,E).

## 4 DISCUSSION

With the discovery of the intracranial glymphatic system and mLVs, research on the links between intracranial lymphatic drainage and central nervous system diseases began to receive increased attention. Studies have shown that neurological diseases are closely related to the function of the intracranial lymphatic system, and that these can be alleviated by improving the function of the glymphatic system and mLVs (Da Mesquita et al., 2018; Pu et al., 2019).

Metabolite clearance in the brain parenchyma depends on both glymphatic system transport and mLVs function. It has been suggested that the glymphatic system helps to clear the breakdown products of various blood components, especially blood clot lysis products, from the CSF–ISF after bleeding (Dreha‐Kulaczewski et al., 2017; Iliff et al., 2012). However, post‐ICH spillage of components within blood vessels and elevated levels of blood cell decomposition products hamper the function of the glymphatic system and choroid plexus, resulting in CSF disorders and various forms of hydrocephalus (Bosche et al., 2020; Gaberel et al., 2014; Macdonald, 2014; Pu et al., 2019). In our study, the glymphatic system was evaluated after ICH based on the clearance of FITC‐dextran and RITC‐dextran from the brain parenchyma and was found to be dysfunctional on day 7 compared with that in normal mice, as the clearance of brain parenchymal RITC‐dextran and FITC‐dextran after ICH was slower compared with that in normal mice. mLVs play an important role in the clearance of metabolic wastes in the CSF. Therefore, evaluating intracranial lymphatic drainage by injecting fluorescent tracers in the cisterna magna is considered a functional assessment of the mLVs. Several recent studies have suggested that the degree of impairment in intracranial mLVs function after chronic subdural hemorrhage varies (Chen et al., 2020; Liu, Gao, et al., 2020). Moreover, the drainage function of mLVs gradually returned to normal levels after ICH in our study, which is also consistent with the findings of another recent study (Tsai et al., 2022).

Our results also showed that rTMS had a significant therapeutic effect—compared with the ICH group, the ICH+rTMS group demonstrated improved glymphatic system and mLVs drainage function and accelerated clearance of brain parenchymal RITC‐dextran and FITC‐dextran. Furthermore, when the bilateral deep cervical lymph vessels were ligated, rTMS‐mediated clearance of RITC‐dextran and FITC‐dextran from the brain parenchyma was halted, indicating that it enhances intracranial lymphatic system drainage and improves the clearance of metabolites from the brain parenchyma after ICH.

Current studies show that arterial pulsation, ICP, anesthesia, and postures can significantly affect the function of the intracranial lymphatic system (Benveniste et al., 2017; Iliff, Wang, et al., 2013; Lee et al., 2015; Lundgaard et al., 2015; Siegel, 2009; Xiang et al., 2022). Therefore, in order to avoid the influence of the above factors on the experimental results, our experiment was conducted in a way to keep the above factors as consistent as possible, for example, the mice were kept in the same anesthesia depth through breathing monitoring during the experiment. A preliminary study in our laboratory shows that the intensity of ICP significantly influenced glymphatic lymphatic fluid transport system (Xiang et al., 2022). In order to clarify whether the changes of ICP after ICH play a role in the improvement of lymphatic drainage by rTMS, ICP was detected in each group on day 7 after rTMS treatment. The results showed that the ICP in ICH and ICH+rTMS groups was significantly higher than that in Sham group; however, there was no significant difference between ICH and ICH+rTMS groups (*p* > .05; Figure S2). Thus, our study concluded that the effect of rTMS on the improvement of intracranial lymphatic drainage after intracerebral hemorrhage was not mediated by changes of ICP. Also, it has been shown that intracranial lymphatic drainage is closely related to the function of the BBB (Boland et al., 2018; Verheggen et al., 2018). The present study further evaluated the changes in the BBB in the ICH and ICH+rTMS groups on day 7 after rTMS treatment, and no statistical difference was found between the two groups. Therefore, our study suggested that the improvement of intracranial lymphatic drainage by rTMS did not work by improving the BBB after ICH.

It has also been suggested that cerebral arterial pulsation is an important driver of paravascular CSF–ISF exchange in the brain (Gaberel et al., 2014), and that changes in arterial pulsation may contribute to the accumulation and deposition of toxic solutes, including amyloid, in the aging brain (Iliff, Wang, et al., 2013). An earlier study directly demonstrated that rTMS increases cortical activity, which is accompanied by changes in vascular hemodynamics (Allen et al., 2007); thus, this may be the underlying mechanism by which rTMS affects the intracranial lymphoid system.

Nevertheless, our current understanding of mLVs and the lymphoid system is in its infancy. Owing to the complex connections between intracranial blood vessels, lymphatic networks, and the peripheral spaces of large vessels, attaining a comprehensive understanding of the glymphatic clearance system in the brain is expected to be a long‐term process. Further research is thus required to ascertain these factors in the treatment of ICH.

### 4.1 Limitations

There are certain limitations of our experimental design that must be considered when interpreting the results of this study. Owing to the species‐specific differences between animals and humans, it is difficult for animal models to truly simulate the relevant pathophysiological changes in human clinical situations, and various animal models have their own scope of application. Additionally, our sample sizes were limited and we only utilized male mice, negating any sex‐related differences in ICH recovery and neuroplasticity.

## 5 CONCLUSION

This study assessed intracranial lymphatic drainage dysfunction after ICH and further explored the effects of rTMS on the regulation of intracranial lymphatic drainage after ICH. Our results show that rTMS stimulation improved the drainage function of the glymphatic system and mLVs in mice with ICH. Thus, rTMS can improve brain parenchymal metabolite clearance after ICH in mice by regulating intracranial lymphatic drainage. These findings could have far‐reaching significance in the context of discovering novel means of regulation for the treatment of ICH. Nevertheless, the potential mechanisms of action still need to be further explored.

## AUTHOR CONTRIBUTIONS

Yuheng Liu, Xuanhui Liu, and Pengju Sun conceived and designed the study and analyzed the data. Zengguang Wang reviewed and revised the manuscript and supervised the study. Jing Li, Meng Nie, Junjie Gong, and Anqi He analyzed the data and interpreted the results. Mingyu Zhao and Chen Yang provided technical support. All authors have read and approved the manuscript.

## CONFLICT OF INTEREST STATEMENT

The authors declare no conflicts of interest.

### PEER REVIEW

The peer review history for this article is available at https://publons.com/publon/10.1002/brb3.3062.

## Supporting information

Fig S1 Statistical steps of glymphatic system.
Fig S2 Changes of ICP in each group on day 7 after treatment.

## Data Availability

The authors confirm that the supporting data for the study are available in the article and from the corresponding author upon reasonable request.
