# Supplementary material for: rTMS treatment for abrogating intracerebral hemorrhage‐induced brain parenchymal metabolite clearance dysfunction in male mice by regulating intracranial lymphatic drainage
Source: Brain Behav. 2023 May 9;13(7):e3062. doi: 10.1002/brb3.3062 (PMC10338767; doi:10.1002/brb3.3062)
Supplement: Supplementary file 1 — Fig S1 Statistical steps of glymphatic system. Fig S2 Changes of ICP in each group on day 7 after treatment. [file BRB3-13-e3062-s001.docx]

**
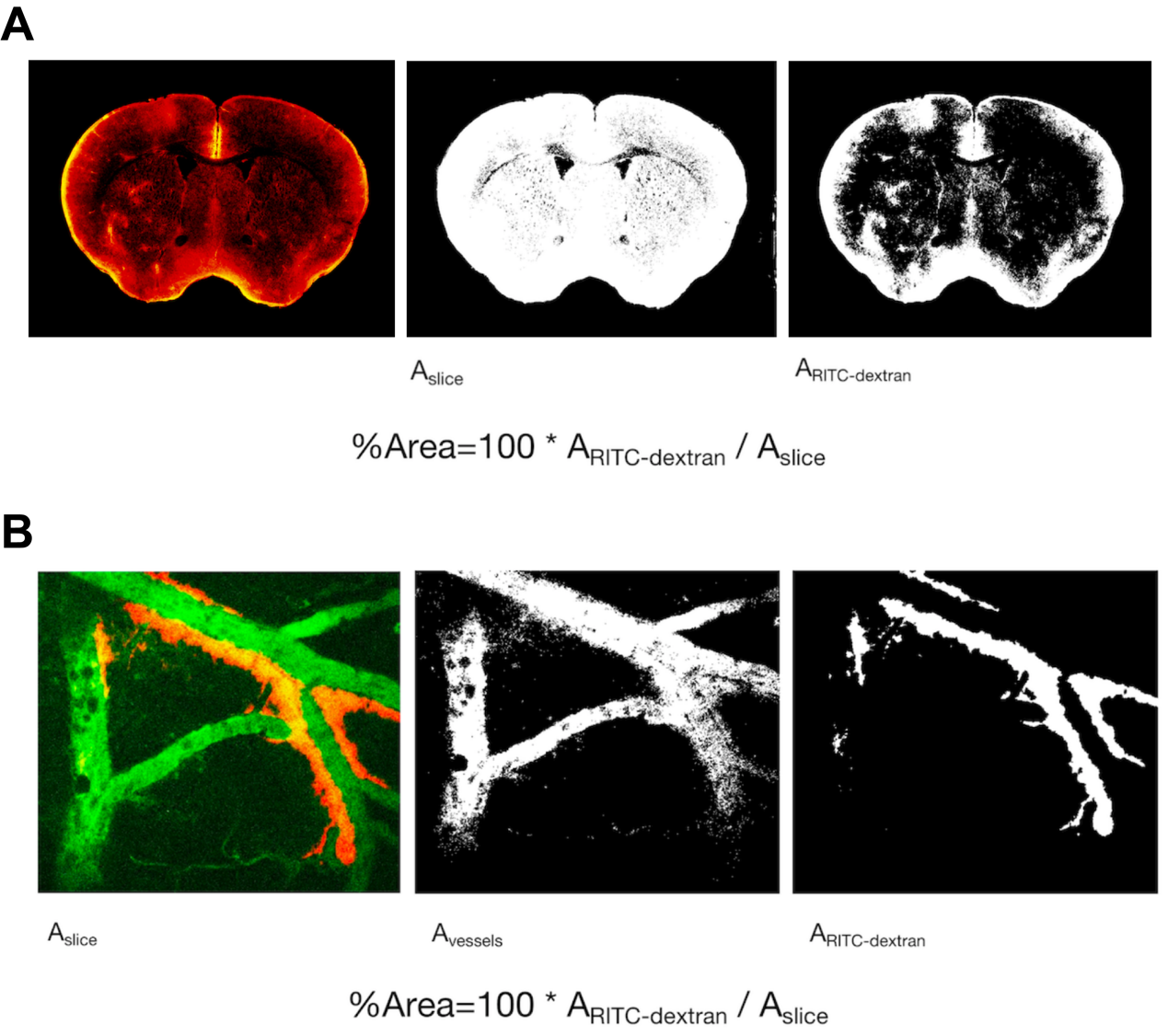
**

**Fig S1 Statistical steps of glymphatic system**

Fig S1 A: After injection of RITC-dextran into the cisterna magna, animals were perfusion fixed and 100-μm vibratome sections cut. Slices were imaged by fluorescence microscopy at 1.25X and montages generated. To evaluate tracer coverage, the color channels were separated, and the backaround subtracted. Thresholded and the thresholded area was calculated and expressed as a percentage of overall slice area.

Fig S1 B: During Two-photon detection, the intensity was quantified using ImageJ software. A weighted average of RITC-dextran fluorescence in the whole region tracers was calculated.


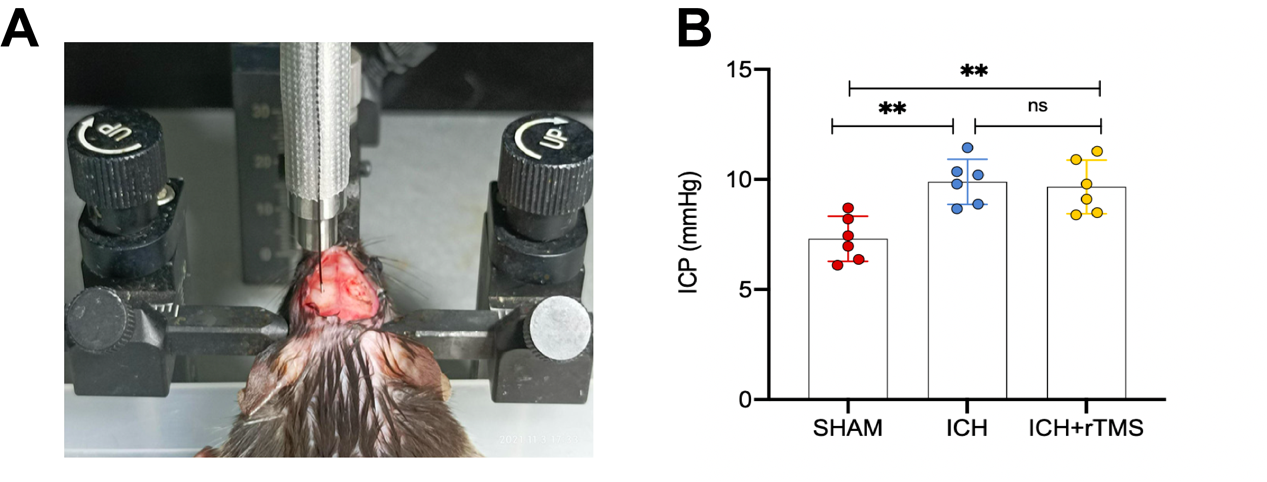


**Fig S2 Changes of ICP in each group on day 7 after treatment**

ICP was detected in each group on the 7th day after rTMS treatment. The results showed that the ICP in ICH and ICH+rTMS groups was significantly higher than that in Sham group, however, there was no significant difference between ICH and ICH+rTMS groups (F (2, 15) = 10.31, n = 9, P = 0.9304). *p < 0.05, **p < 0.01, ***p < 0.001, ****p < 0.0001.
